# *Acanthus ebracteatus* leaf extract provides neuronal cell protection against oxidative stress injury induced by glutamate

**DOI:** 10.1186/s12906-018-2340-4

**Published:** 2018-10-16

**Authors:** Anchalee Prasansuklab, Tewin Tencomnao

**Affiliations:** 1 Graduate Program in Clinical Biochemistry and Molecular Medicine, Department of Clinical Chemistry, Faculty of Allied Health Sciences, Chulalongkorn University, Bangkok, 10330 Thailand; 2 Age-Related Inflammation and Degeneration Research Unit, Department of Clinical Chemistry, Faculty of Allied Health Sciences, Chulalongkorn University, Bangkok, 10330 Thailand

**Keywords:** *Acanthus ebracteatus*, HT22 cells, Glutamate toxicity, Oxidative stress, Oxytosis, Neuroprotection, Antioxidant, Nrf2/ARE pathway

## Abstract

**Background:**

*Acanthus ebracteatus* (AE), an herb native to Asia, has been recognized in traditional folk medicine not only for its antioxidant properties and various pharmacological activities but also as an ingredient of longevity formulas. However, its anti-neurodegenerative potential is not yet clearly known. This work aimed to evaluate the protective effect of AE leaf extract against glutamate-induced oxidative damage in mouse hippocampal HT22 cells, a neurodegenerative model system due to a reduction in glutathione levels and an increase in reactive oxygen species (ROS).

**Methods:**

Cell viability, apoptosis, and ROS assays were performed to assess the protective effect of AE leaf extract against glutamate-induced oxidative toxicity in HT22 cells. The antioxidant capacity of AE was evaluated using in vitro radical scavenging assays. The subcellular localization of apoptosis-inducing factor (AIF) and the mRNA and protein levels of genes associated with the nuclear factor erythroid 2–related factor 2 (Nrf2) antioxidant system were determined to elucidate the mechanisms underlying the neuroprotective effect of AE leaf extract.

**Results:**

We demonstrated that AE leaf extract is capable of attenuating the intracellular ROS generation and HT22 cell death induced by glutamate in a concentration-dependent manner. Co-treatment of glutamate with the extract significantly reduced apoptotic cell death via inhibition of AIF nuclear translocation. The increases in Nrf2 levels in the nucleus and gene expression levels of antioxidant-related downstream genes under Nrf2 control were found to be significant in cells treated with the extract.

**Conclusions:**

The results suggested that AE leaf extract possesses neuroprotective activity against glutamate-induced oxidative injury and may have therapeutic potential for the treatment of neurodegenerative diseases associated with oxidative stress.

## Background

Oxidative stress is classically described as an imbalance of redox homeostasis, resulting from the overproduction of free radicals relative to the innate ability of cells to scavenge them. This detrimental event causes damage to cellular components and alterations in cellular function that ultimately contribute to cell death [1, 2]. Reactive oxygen species (ROS) are the most common type of free radicals produced in the human body and play an important role in cellular injury in various tissues, particularly the central nervous system (CNS), which is highly sensitive to oxidative damage due to its large dependence on oxygen consumption [3, 4]. In fact, oxidative stress is associated with aging and is a common pathological feature of age-related neurodegenerative diseases, such as Alzheimer’s disease (AD), Parkinson’s disease (PD), and amyotrophic lateral sclerosis (ALS), in which ROS accumulation is implicated in the mechanism of neuronal loss [5–7]. Currently, many researchers believe that compounds or drugs possessing powerful antioxidant activity could be effective in treating such ROS-related diseases [8–10].

Glutamate, the principal excitatory neurotransmitter in the brain, has been suggested as a critical trigger of neuronal cell death in several CNS disorders and neurodegenerative diseases [11]. In addition to its involvement in many aspects of normal brain functions, glutamate can also act as a neurotoxin when it is present in excessively high concentrations in the brain extracellular space, causing cellular damage in the context of neurodegeneration. One of the main mechanisms underlying the neurotoxic effects of glutamate is an oxidative stress-induced programmed cell death pathway called oxytosis [12]. In this cell death paradigm, glutamate at pathological levels induces inhibition of cystine uptake via the cystine/glutamate antiporter (system Xc^−^), leading to impaired production of the endogenous antioxidant glutathione (GSH) and thereby enhancing accumulation of ROS as well as oxidative stress. Subsequently, the elevated ROS level disrupts mitochondrial membrane integrity and results in the release of apoptosis-inducing factor (AIF), which eventually triggers neuronal death via a caspase-independent pathway [13, 14]. Therefore, suppression of glutamate-induced oxidative stress-mediated neuronal cell death may have the potential to provide a beneficial therapeutic approach for the treatment of neurodegenerative diseases.

At the present time, there is growing worldwide use of herbal medicines for preventive and therapeutic applications based on historical knowledge. In connection with the aforementioned mechanism of glutamate-induced oxidative toxicity, medicinal plants or naturally derived compounds with antioxidant and antiapoptotic effects are currently being researched as neuroprotective agents [15–19]. *Acanthus ebracteatus* Vahl. (AE), commonly known as “Sea Holly”, is a medicinal mangrove plant in the family Acanthaceae and is widely distributed in Southeast Asia, including China, India, and Australia [20, 21]. All parts of this plant have been used historically for a variety of medicinal purposes, such as hair root nourishment, reduction of cough and fever, expulsion of kidney stones, relief of rheumatoid arthritis pain and inflammation, and treatment of hypertension, cancer, skin diseases such as rash, chronic wounds and snakebites [22–26]. Interestingly, AE is also used as an important ingredient in traditional Thai longevity and neurotonic remedies for improving brain and body functions [23, 27]. Moreover, previous chemical investigations on this plant revealed the presence of some bioactive components possessing considerable antioxidant activity, neuromodulatory function or memory-improving effects [28–32]. However, currently, there is no conclusive evidence to substantiate its brain and neural health promotion properties. Thus, the present study was conducted to investigate, for the first time, the neuroprotective effect of AE leaf extract against glutamate-induced oxidative cytotoxicity and to further elucidate its underlying protective mechanisms using the mouse hippocampal neuronal HT22 cell line as a cellular model of neurodegeneration.

## Methods

### Plant material and preparation of the extracts

The plant material used in this study is the leaves of *A. ebracteatus* collected from the Princess Maha Chakri Sirindhorn Herbal Garden (Rayong Province, Thailand). The plant was authenticated by Professor Dr. Thaweesakdi Boonkerd and deposited with voucher specimen number A013422(BCU) at the herbarium of Kasin Suvatabhandhu (Department of Botany, Faculty of Science, Chulalongkorn University, Thailand). The extraction was carried out twice using hexane and absolute ethanol as extracting solvents. Briefly, the leaves were dried in a ventilated incubator at a temperature of at 40 °C and ground into a fine powder. Then, the extracts were prepared by macerating 35 g of the dried leaf powder in 350 mL of each solvent for 48 h under agitation at room temperature (RT), followed by filtration. The residue powder was re-extracted by a similar process, and all filtrates were subsequently combined before removing the solvent by vacuum evaporation. The yield of hexane extract (AEH) and ethanolic extract (AEE) of *A. ebracteatus* leaves was found to be 2.14% and 7.98% (*w*/w), respectively. Each resulting extract was dissolved in dimethyl sulfoxide (DMSO) as a stock solution of 100 mg/mL, stored at − 20 °C, and protected from light until further analysis.

### Determination of total flavonoid content

The total flavonoid content was determined using the aluminum chloride colorimetric method modified for a microplate format as described previously [33]. In brief, 50 μL of the extract sample (1 mg/mL) was made up to 200 μL with 95% ethanol, and mixed well with 10 μL of 10% (*v*/v) AlCl_3_ solution and 10 μL of 1 M NaOAc solution. After the reaction was allowed to stand for 40 min in the dark, the absorbance of the reaction mixture was measured at 415 nm using a microplate reader (Perkin-Elmer). Quercetin (Sigma-Aldrich) was used as a standard to construct the calibration curve for quantification, and the content of total flavonoids was reported as mg of quercetin equivalent (QE) per g of dry weight extract.

### Determination of total phenolic content

The total phenolic content was determined using the Folin-Ciocalteu method adapted for analysis with a microplate reader, as previously described [33]. Briefly, 50 μL of the extract sample (1 mg/mL) was mixed thoroughly with 50 μL of 10-fold diluted Folin-Ciocalteu’s phenol reagent (Sigma-Aldrich). After 20 min of incubation, the mixture was neutralized by addition of 50 μL of a 7.5% (*w*/*v*) Na_2_CO_3_ solution and then kept in the dark at RT for a further 20 min. Finally, the absorbance was measured at 760 nm using an EnSpire® Multimode Plate Reader (Perkin-Elmer). The content of total phenolics was calculated from a standard calibration curve using gallic acid (TCI America, Portland, OR, USA), and the results are expressed as mg of gallic acid equivalent (GAE) per g of dry weight extract.

### LC-MS analysis

The extract was submitted to Institute of Systems Biology (Universiti Kebangsaan Malaysia, Malaysia) for screening of phytochemical constituents using Liquid Chromatography-Mass Spectrometry (LC-MS) analysis. The analytical system used was a Dionex™ UltiMate 3000 UHPLC system (Thermo Fisher Scientific) coupled with a high-resolution micrOTOF-Q III (Bruker Daltonik GmbH, Bremen, Germany). The chromatographic separation was performed on an Acclaim™ Polar Advantage II C18 column (3 μm, 3 mm × 150 mm) (Thermo Fisher Scientific) with a gradient mobile phase consisting of 0.1% formic acid in water (A) and 100% acetonitrile (B). The elution program was as follows: 5% B (0-3 min); 80% B (3-10 min); 80% B (10-15 min) and 5% B (15-22 min). The flow rate was 400 μL/min and the injection volume was 1 μL. The MS instrument was operated in the positive electrospray ionization (ESI) mode with the parameters setting as follows: drying gas flow at 8 L/min, drying gas temperature at 200 °C, nebulizer pressure at 1.2 bar, capillary voltage at 4500 V, and m/z scan range of 50 to 1000. For identification of putative compounds, the observed (experimental) m/z values were compared with the METLIN and the KNApSAcK databases as well as with the calculated (theoretical) mass values of previously reported compounds in *A. ebracteatus*, with an accepted difference of less than 30 parts-per-million (ppm). Relative amount is expressed as the percentage of peak area relative to total area of all peaks observed in the chromatogram.

### Cell culture

The immortalized mouse hippocampal HT22 cell line, which served as an in vitro model of neurodegeneration, was a generous gift from Prof. David Schubert at the Salk Institute (San Diego, CA, USA). The HT22 cell line was originally a glutamate-sensitive subclone of the HT-4 cell line, which was derived from the immortalization of primary mouse hippocampal neuronal tissues with a temperature-sensitive SV40 T-antigen [34]. These cells were cultured in Dulbecco’s modified Eagle’s medium (DMEM) (Sigma-Aldrich, St. Louis, MO, USA) supplemented with 10% (*v*/v) fetal bovine serum (Sigma-Aldrich), 100 units/mL penicillin, and 100 μg/mL streptomycin (Gibco, Waltham, MA, USA) in a humidified atmosphere of 5% CO_2_ at 37 °C. The culture medium was replaced every two days. Upon reaching approximately 80% confluency, the cells were passaged by trypsinization and subcultured into fresh medium to maintain their exponential growth.

### MTT reduction assay

The MTT assay measures cell viability based on the metabolic activity of viable cells to reduce the yellow tetrazolium salt, 3-(4,5-dimethylthiazol-2-yl)-2,5-diphenyltetrazolium bromide (MTT), to a purple formazan product. HT22 cells were seeded at a density of 6 × 10^3^ cells/well in a 96-well plate and incubated overnight prior to treatment with 5 mM glutamate (Sigma-Aldrich) alone or glutamate in combination with different concentrations of the extracts for 24 h. After the exposure period, the MTT solution (Biobasic, Markham, Ontario, Canada) was added to each well at a final concentration of 0.5 mg/mL and incubated for an additional 4 h in the dark. The generated formazan crystals were dissolved in a DMSO-ethanol mixture (1:1, *v*/v) after the supernatants were carefully removed from the wells. Finally, the absorbance was determined at 550 nm by using an EnSpire® Multimode Plate Reader (Perkin-Elmer, Waltham, MA, USA). The results are expressed as a percentage relative to control (untreated) cells.

### LDH leakage assay

The LDH assay determines cell viability based on the release of cytoplasmic lactate dehydrogenase (LDH) from damaged cells, which converts the colorless tetrazolium salt, 2-(*p*-iodophenyl)-3-(*p*-nitrophenyl)-5-phenyl tetrazolium chloride (INT), into a red formazan product. HT22 cells were seeded at a density of 6 × 10^3^ cells/well in a 96-well plate and incubated overnight prior exposure to 5 mM glutamate alone or glutamate in combination with different concentrations of the extracts for 24 h. After treatment, the amount of LDH released into the culture medium was measured using the CytoTox 96® assay (Promega, Madison, WI, USA) according to the manufacturer’s instructions. In brief, the culture supernatant was incubated with reconstituted substrate mix in the dark for 30 min at RT, followed by addition of the stop solution before measurement. The absorbance was then recorded at 490 nm by a microplate reader (Perkin-Elmer). The results are expressed as a percentage of maximum LDH release obtained by complete lysis of control (untreated) cells.

### Flow cytometric determination of apoptotic cells

Apoptotic cell death was determined based on the externalization of phosphatidylserine to the outer cell surface and increased plasma membrane permeability to dye by using an FITC Annexin V apoptosis detection kit with propidium iodide (PI) (BioLegend, San Diego, CA, USA) according to the manufacturer’s protocol. Briefly, HT22 cells were seeded onto a 6-well plate at a density of 1.5 × 10^5^ cells/well and incubated overnight prior to treatment with 5 mM glutamate alone or glutamate in combination with the extracts for 18 h. At the end of the exposure period, the harvested cells were washed twice with phosphate-buffered saline (PBS), re-suspended in the binding buffer, and stained by the solution of FITC-conjugated annexin V and PI for 15 min in the dark. The fluorescence intensity of stained cells, at least 10,000 cells per group, was immediately analyzed by a BD FACSCalibur™ flow cytometer (BD Bioscience, Heidelberg, Germany). The results are expressed as a percentage of annexin V-positive/PI-negative cells (early apoptosis) plus annexin V/PI-positive cells (late apoptosis).

### In vitro radical scavenging assay

The radical scavenging assay was performed using the DPPH and ABTS methods for evaluation of antioxidant activity of the sample based on its hydrogen atom- or electron-donating capacity to neutralize the free radicals. A working solution of stable free radical 2,2-diphenyl-1-picrylhydrazyl (DPPH•) (Sigma-Aldrich) was dissolved in ethanol to a final concentration of 0.2 mg/mL. The cation radical ABTS• + working solution was generated by the oxidation of 7 mM 2,2′-azinobis-(3-ethylbenzothiazoline-6-sulfonic acid) (ABTS) (Sigma-Aldrich) with 2.45 mM potassium persufhate at a 1:1 (*v*/v) ratio. The reaction mixture was allowed to stand for 16–18 h in the dark prior to dilution with ethanol until the absorbance reached between 0.7 and 0.8 at 734 nm. For the assay protocol, the DPPH• or ABTS• + working solution was added to the extract sample (1 mg/mL) at a ratio of 9:1 (*v*/v). The reaction mixture was incubated in the dark at RT for 15 min or 30 min, and the absorbance was recorded using a microplate reader (Perkin-Elmer) at 517 nm or 734 nm for the DPPH or ABTS assay, respectively. Ascorbic acid (vitamin C) (Calbiochem, San Diego, CA, USA) was used as a reference standard in both assays. Radical scavenging activity was expressed as the percent inhibition of free radicals calculated by the following equation: % Inhibition = 100 - [(Abs of sample - Abs of blank) × 100/ Abs of control]. The antioxidant capacity is expressed as vitamin C equivalent antioxidant capacity (VCEAC) in mg per g of dry weight extract.

### Assay for intracellular ROS level

Measurement of the intracellular ROS level was performed using oxygen-sensitive 2′,7′-dichloro-dihydrofluoroscein diacetate (H_2_DCFDA) (Molecular Probes, Eugene, OR, USA) based on the ability of ROS to oxidize the cell-permeant, non-fluorescent H_2_DCFDA molecule into a highly fluorescent 2′,7′-dichlorofluorescein (DCF) molecule. HT22 cells were seeded at a density of 1 × 10^4^ cells/well in a 96-well plate and incubated overnight prior to exposure to 5 mM glutamate alone or glutamate in combination with different concentrations of the extracts for 14 h. After treatment, the cells were loaded with 5 μM H_2_DCFDA, incubated at 37 °C for another 30 min, and then washed three times with Hank’s balanced salt solution (HBSS) (Gibco). Fluorescence was immediately determined using an EnSpire® Multimode Plate Reader (Perkin-Elmer), and photographs were obtained using an Axio Observer A1 fluorescence microscope (Carl Zeiss, Jena, Germany), with an excitation wavelength of 485 nm and an emission wavelength of 535 nm. Data are expressed as the percentage of fluorescence intensity relative to control (untreated) cells.

### Quantitative real-time PCR analysis

At the end of treatments, the total RNA from HT22 cells in each group was extracted with Trizol reagent (Invitrogen, Carlsbad, CA, USA) according to the manufacturer’s instructions. The amount of RNA was quantified by measuring absorbance at 260 nm, and then 1 μg of total RNA was reverse transcribed to cDNA using oligo(dT)17 primer and AccuPower RT PreMix (Bioneer, Daejeon, South Korea). The cDNA was used as a template for subsequent real-time PCR reactions performed on an Exicycler™ 96 real-time quantitative thermal block (Bioneer) by using the GreenStar™ qPCR PreMix (Bioneer) and the specific primers listed in Table 1. The thermal cycling conditions included an initial denaturation step at 95 °C for 10 min, followed by 40 cycles, each consisting of 15 s of denaturation at 95 °C, 15 s at 55 °C for primer annealing, and 30 s at 72 °C for chain elongation. A melting curve analysis was performed after amplification to verify the accuracy of the amplicon. The relative expression level of each target gene was normalized to β-actin expression and analyzed using the 2^-ΔΔCT^ method.

**Table 1:** Primers used for real-time PCR

Gene	Accession number	Sequence (5' → 3')	Product length (bp)
EAAT3	NM_009199.2	Forward: ATGATCTCGTCCAGTTCGGC	202
		Reverse: TGACGATCTGCCCAATGCTT	
NQO1	NM_008706.5	Forward: CGACAACGGTCCTTTCCAGA	253
		Reverse: CTCCCAGACGGTTTCCAGAC	
GCLM	NM_008129.4	Forward: GGAGCTTCGGGACTGTATCC	236
		Reverse: CAACTCCAAGGACGGAGCAT	
ACTB	NM_007393.5	Forward: GGCTGTATTCCCCTCCATCG	154
		Reverse: CCAGTTGGTAACAATGCCATGT	

### Western blot analysis

Protein expression of target genes was determined by Western blotting. After the treatments, HT22 cells were harvested, washed, and prepared for whole cell lysates as well as cytoplasmic and nuclear fractions. Whole cell lysates were obtained by lysing the cells on ice in NP-40 lysis buffer (50 mM Tris pH 8.0, 150 mM NaCl, 1% NP-40, 1 mM PMSF, 1 mM DTT). Cytoplasmic and nuclear fractions were isolated using the NE-PER nuclear and cytoplasmic extraction reagents (Thermo Fisher Scientific, Rockford, IL, USA) according to the manufacturer’s protocol. Total protein concentrations were measured by the Bradford reagent (Bio-Rad, Hercules, CA, USA) with bovine serum albumin (BSA) as a standard. Equal amounts of proteins were separated by electrophoresis on 10% (*v*/v) SDS-polyacrylamide gels and electrotransferred to polyvinylidene difluoride (PVDF) membranes. The membranes were blocked for 1 h with 5% skim milk in TBS-T (Tris-buffered saline, 0.1% Tween 20) and allowed to incubate overnight at 4 °C with primary antibodies specific for nuclear factor erythroid 2-related factor 2 (Nrf2) (1:2000; Santa Cruz Biotechnology, Dallas, Texas, USA), excitatory amino acid transporter 3 (EAAT3) (1:8000; Abcam, Cambridge, UK), apoptotic-inducing factor (AIF) (1:2000; Cell Signaling Technology, Danvers, MA, USA), Lamin B1 (1:2000; Cell Signaling Technology) or β-actin (1:16000; Cell Signaling Technology) and subsequently incubated for an additional 45 min at RT with horseradish peroxidase (HRP)-conjugated secondary antibodies (1:10000; Cell Signaling Technology). Specific protein bands were visualized with an enhanced chemiluminescence (ECL) detection reagent (GE Healthcare, Marlborough, MA, USA). Densitometric analysis of the bands was performed with an image analysis system (Syngene, Cambridge, UK).

### Immunofluorescence microscopy

HT22 cells were seeded in 12-well plate at a density of 4 × 10^4^ cells/well prior exposure to 5 mM glutamate alone or glutamate in combination with the extract for 16 h. After treatments, an immunofluorescence technique was performed to determine nuclear translocation of AIF. In brief, the cells were fixed with cold 4% (*w*/*v*) paraformaldehyde solution for 20 min, permeabilized in 0.1% (w/v) Triton X-100 for 10 min, and blocked with 5% (w/v) BSA for 30 min. Then, the cells were incubated overnight with anti-AIF antibody (1:400; Cell Signaling Technology) at 4 °C, followed by incubation for 1 h with Alexa Fluor 555-conjugated goat anti-rabbit (1:2000; Sigma-Aldrich) at RT. Nuclei were counterstained with 4′,6-diamidino-2-phenylindole (DAPI) solution for 10 min at RT. Following mounting with ProLong Gold antifade mountant (Thermo Fisher Scientific), stained cells were imaged using an LSM 700 confocal laser scanning microscope (Carl Zeiss, Jena, Germany).

### Statistical analysis

All experiments were performed in at least triplicate and the data are represented as means ± standard deviation (SD) or means ± standard error of mean (SEM) as indicated in figures. All of the calculations were performed using SPSS software version 17.0 (SPSS Inc., Chicago, IL, USA). The differences between groups were analyzed using one-way analysis of variance (ANOVA), followed by the post hoc Tukey HSD multiple comparison test. The results were considered statistically significant when *P* < 0.05.

## Results

### Analysis of phytochemical compounds in AEE

**Fig. 1:**
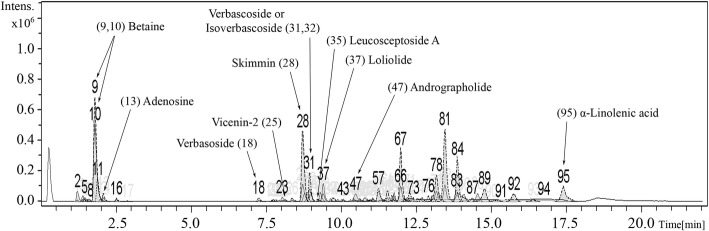
LC-MS total ion chromatogram of AEE obtained in positive ESI mode. All indicated peak numbers of proposed compounds are detailed in Table 2

To identify putative phytochemical components in AE leaf extract that may be responsible for neuroprotection against glutamate-induced oxidative toxicity, we carried out LC-MS analysis as well as quantitative determination of total flavonoids and phenolics in AEE. Our results revealed a total of 95 ion chromatographic peaks of AEE detected in the positive ion mode (Fig. 1). After identification of each molecular ion peak [M + H]^+^ by comparison of observed m/z values with the calculated (theoretical) values recorded in databases and the literature, we proposed 11 phytochemical compounds that could have beneficial effects for antioxidant defense or neurological function, of which 5 have been previously reported for *A. ebracteatus* (Fig. 1 and Table 2). The identified peaks were annotated by number and are detailed in Table 2 as follows: peak number, retention time (Rt), observed m/z, peak area, compound name, theoretical mass, and mass error. Furthermore, the total flavonoid content and total phenolic content of AEE were found to be 20.22 ± 3.69 mg QE and 84.86 ± 3.69 mg GAE per g of dry weight extract, respectively.

**Table 2:** Proposed phytochemical constituents in AEE

Peak No.	Rt (min)	[M + H]^+^ (m/z)	Area (%)	Proposed compound	Theoretical mass	Mass error (ppm)	Database/ Reference
9	1.8	118.088	10.5	Betaine	117.079	14	METLIN
10	1.8	118.087	7.7	Betaine	117.079	6	METLIN
13	2.1	268.104	0.7	Adenosine	267.097	0	METLIN, [23]
18	7.3	463.181	0.4	Verbasoside	462.174	0	METLIN
25	8.1	595.165	0.2	Vicenin-2	594.158	1	METLIN, [23]
28	8.7	325.091	6.9	Skimmin	324.085	2	METLIN, KNApSAcK
31	9.0	625.212	2.3	Verbascoside, Isoverbascoside	624.205	1	METLIN, [23]
32	9.0	625.210	1.2	Verbascoside, Isoverbascoside	624.205	4	METLIN, [23]
35	9.2	639.228	0.2	Leucosceptoside A	638.221	1	[23]
37	9.4	197.117	1.7	Loliolide	196.110	1	METLIN, KNApSAcK
47	10.5	351.214	1.1	Andrographolide	350.209	7	METLIN
95	17.4	279.231	2.9	α-Linolenic Acid	278.225	3	METLIN

### AE leaf extracts attenuate glutamate-induced cytotoxicity in HT22 cells

**Fig. 2:**
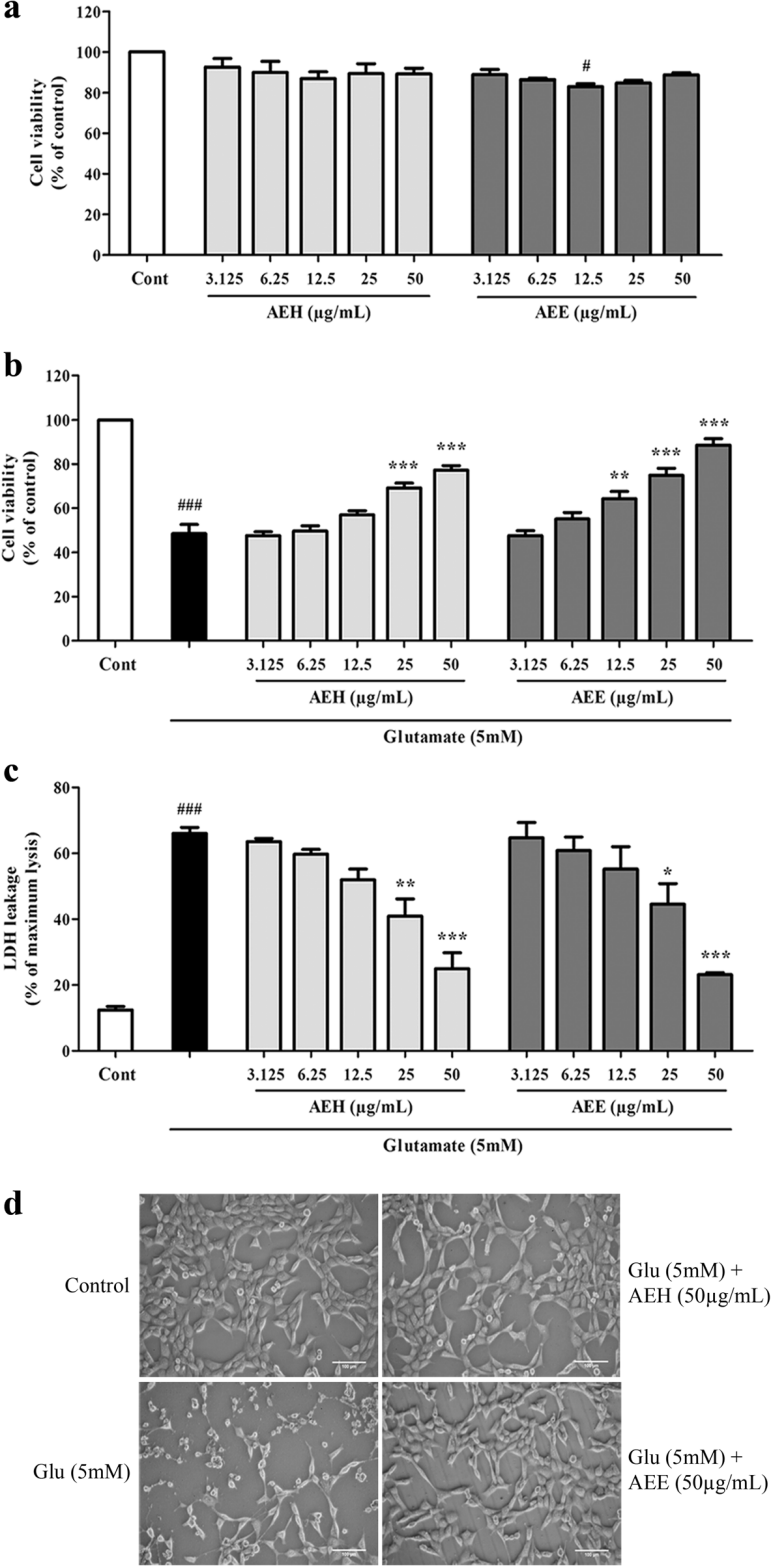
Protective effect of AE leaf extracts against glutamate-induced cytotoxicity in HT22 cells. (**a**) Relative MTT viability of HT22 cells exposed to various concentrations of AEH or AEE. (**b**) Relative MTT viability of HT22 cells exposed to 5 mM glutamate alone or glutamate combined with different concentrations of extracts for 24 h. (**c**) Relative LDH release from HT22 cells exposed to similar treatment conditions as in (**b**). (**d**) Representative morphological images at 5X magnification of untreated HT22 cells (control), or cells treated with glutamate alone or with glutamate plus either AEH or AEE at 50 μg/mL. Data are expressed as the means ± SEM, ^*#*^*P* < 0.05, ^*###*^*P* < 0.001 vs. control; *P < 0.05, **P < 0.01, ***P < 0.001 vs. glutamate alone

To examine the neuroprotective effect of AE leaf extracts against glutamate-induced oxidative cytotoxicity, HT22 cells were exposed to various concentrations of either AEE or AEH (3.125, 6.25, 12.5, 25, and 50 μg/mL) in the presence or absence of 5 mM glutamate, and then cell viability was assessed. We first evaluated the toxic effects of AEH and AEE on HT22 cells and found that neither extract caused noticeable cell death, as shown in Fig. 2a. Treatment of glutamate alone at 5 mM caused a reduction in cell viability of approximately 50%; however, this effect could be rescued in the presence of AE leaf extracts. Our results showed that both AEH and AEE exhibited a significant protective effect by restoring the viability of glutamate-treated HT22 cells in a dose-dependent manner, as determined by the MTT reduction (Fig. 2b) and LDH leakage (Fig. 2c) assays. Morphological examination under a microscope showed normal morphology of HT22 cells upon glutamate treatment combined with AEH or AEE (Fig. 2d). These results indicate that AE leaf extracts protect against neuronal damage caused by glutamate. The concentration of 50 μg/mL AEH and AEE was chosen for subsequent experiments, as it resulted in maximal protection among the concentrations tested.

### AE leaf extracts suppress glutamate-induced apoptosis in HT22 cells

**Fig. 3:**
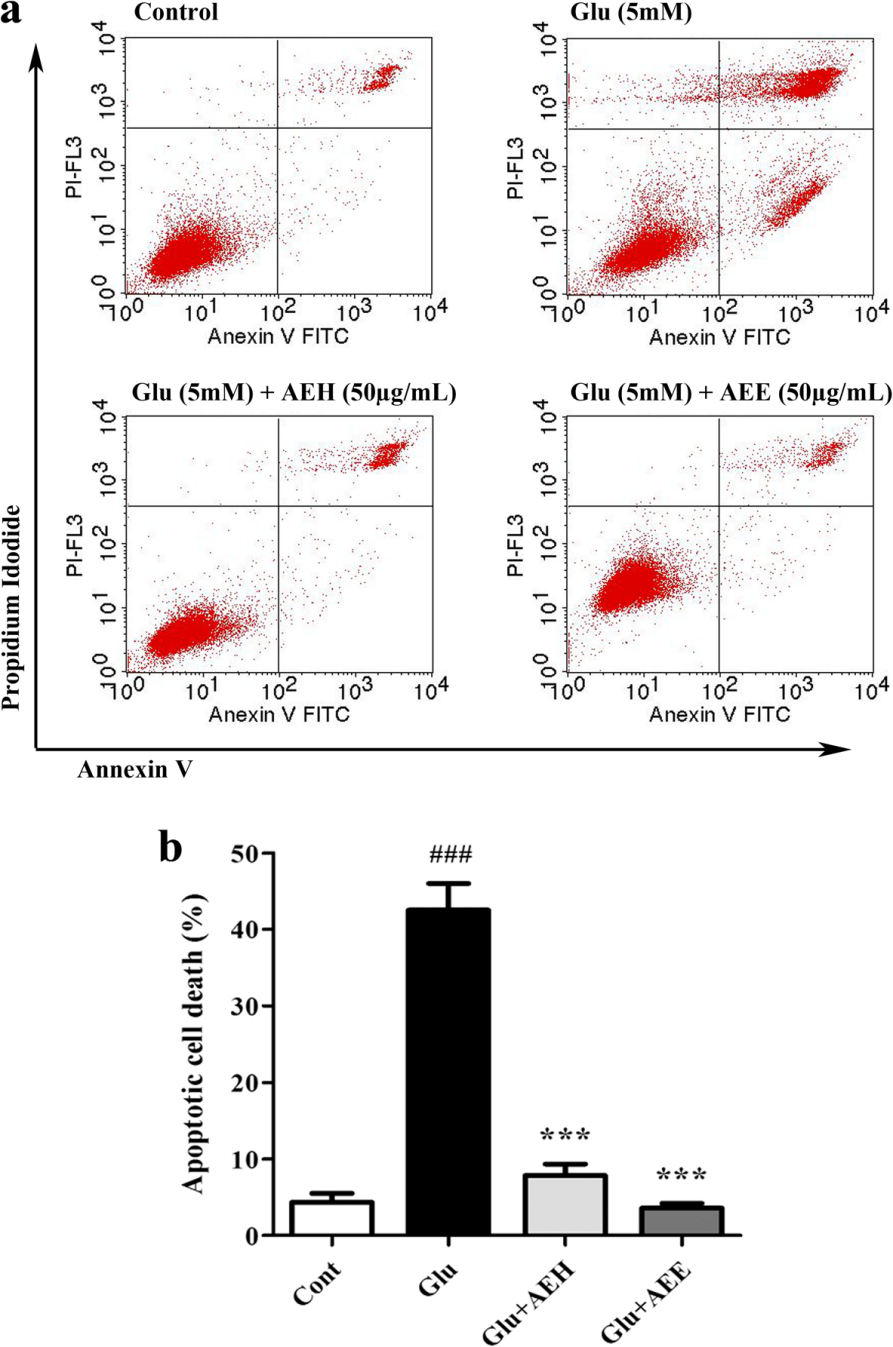
Protective effect of AE leaf extracts against glutamate-induced apoptotic cell death in HT22 cells. (**a**) Representative flow cytometric scatter plots of annexin V-FITC and PI staining in untreated HT22 cells (control), or cells exposed to 5 mM glutamate alone or glutamate combined with either AEH or AEE at 50 μg/mL for 18 h. (**b**) The percentages of apoptotic cells in each treatment group calculated as the sum of annexin V-positive/PI-negative cells (early-stage apoptosis, lower right quadrant) plus annexin V/PI double-positive cells (late-stage apoptosis, upper right quadrant). Data are expressed as the means ± SD, ^*###*^*P* < 0.001 vs. control; ***P < 0.001 vs. glutamate alone

As it is known that the toxicity caused by excessive glutamate contributes to neuronal cell death via the apoptotic pathway [12], we therefore tested whether the AE leaf extracts could suppress glutamate-induced apoptotic cell death by using Annexin V-FITC/PI staining and flow cytometric analysis to further confirm the neuroprotective effect of the extracts. Our results demonstrated the induction of apoptosis in HT22 cells following glutamate exposure and that the percentage of apoptotic cell death in 5 mM glutamate-treated cells was dramatically increased to approximately 40% compared to that of the control, in which the majority of apoptotic cells were in late stage (Fig. 3a). However, co-treatment of the cells with 50 μg/mL AEH or AEE could significantly reduce the apoptotic rate of glutamate-treated cells to an extent comparable to that observed in the control cells (Fig. 3b), indicating the cytoprotective and antiapoptotic effects of AE leaf extracts against glutamate toxicity in neurons.

### AE leaf extract inhibits glutamate-induced AIF nuclear translocation in HT22 cells

**Fig. 4:**
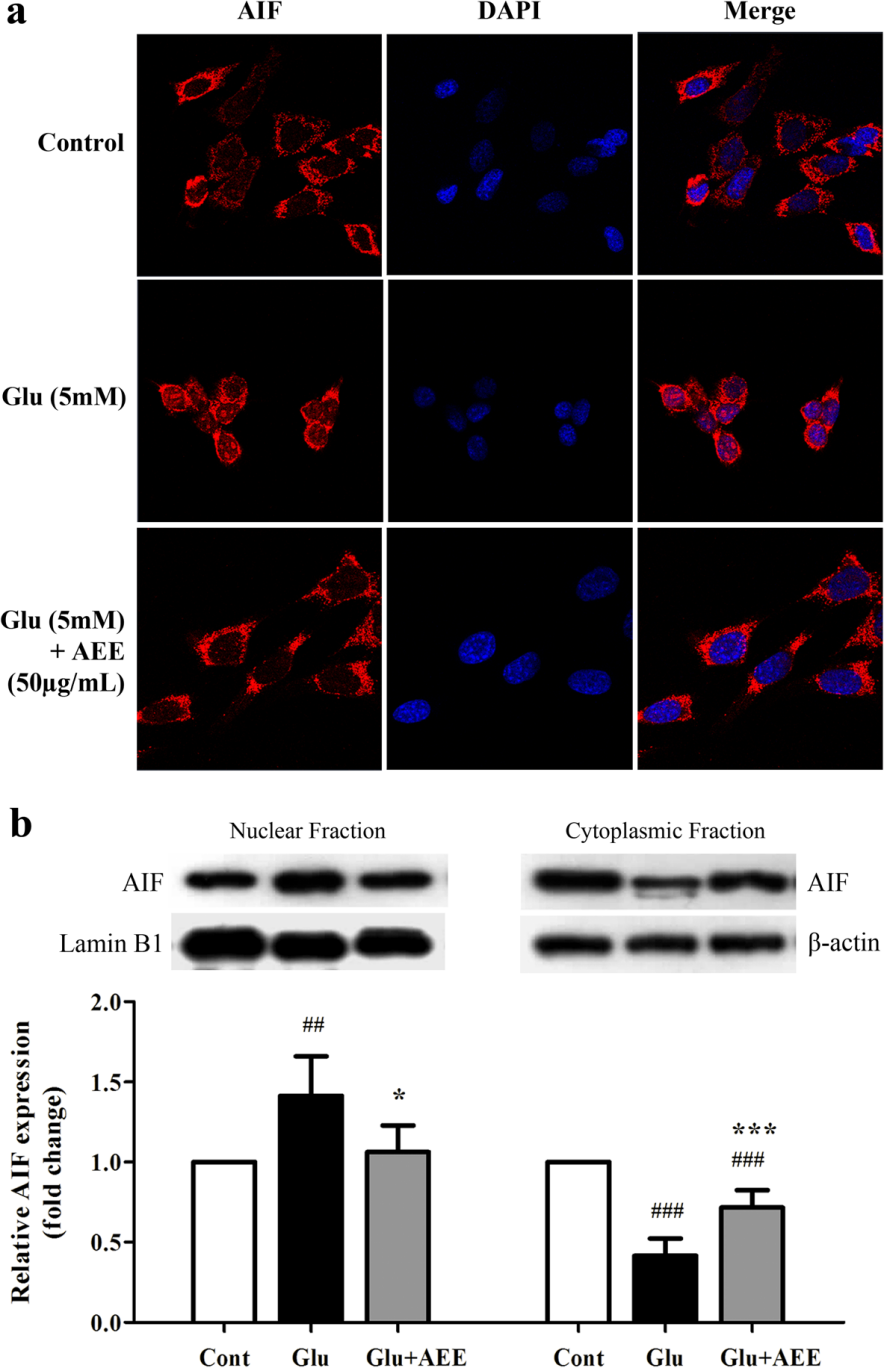
Effect of AE leaf extract on subcellular distribution of AIF in glutamate-treated HT22 cells. (**a**) Representative confocal photographs of immunofluorescence staining with an antibody specific for AIF (red) and nuclei counterstaining with DAPI (blue) of untreated HT22 cells (control; top panel) or cells exposed to 5 mM glutamate alone (middle panel) or glutamate combined with 50 μg/mL AEE (bottom panel) for 16 h. (**b**) Western blot analysis of AIF protein in nuclear and cytoplasmic fractions isolated from HT22 cells exposed to the similar treatment conditions as in (**a**). Lamin B1 and β-actin were used as endogenous loading controls to normalize the expression level of AIF protein from nuclear and cytoplasmic fractions, respectively. Data are expressed as the means ± SD, ^*##*^*P* < 0.01, ^*###*^*P* < 0.001 vs. control; *P < 0.05, ***P < 0.001 vs. glutamate alone

Previous studies have demonstrated that the main mechanism of glutamate-induced apoptotic cell death in HT22 cells is mediated by AIF translocation into the nucleus [35]. Thus, we performed immunocytochemistry and Western blot analysis to determine the effect of AE leaf extract on the subcellular distribution of AIF. Our results revealed that AIF proteins, which are mainly distributed throughout the cytosol under control conditions, were translocated into neuronal nuclei of the HT22 cells following treatment with 5 mM glutamate (Fig. 4a). Moreover, the AIF expression detected by Western blotting was found to be significantly increased in the nucleus but decreased in the cytoplasm (Fig. 4b), confirming that glutamate caused the nuclear translocation of AIF. However, exposure of glutamate-treated cells to 50 μg/mL of AEE significantly restored both nuclear and cytoplasmic expression of AIF proteins to a level similar to those of untreated cells (Fig. 4a and b). These results suggest that the protective effect of AE leaf extract against neuronal cell death may be mediated by the inhibition of glutamate-induced translocation of AIF to the nucleus.

### AE leaf extracts protects neurons against glutamate-induced oxidative stress

**Fig. 5:**
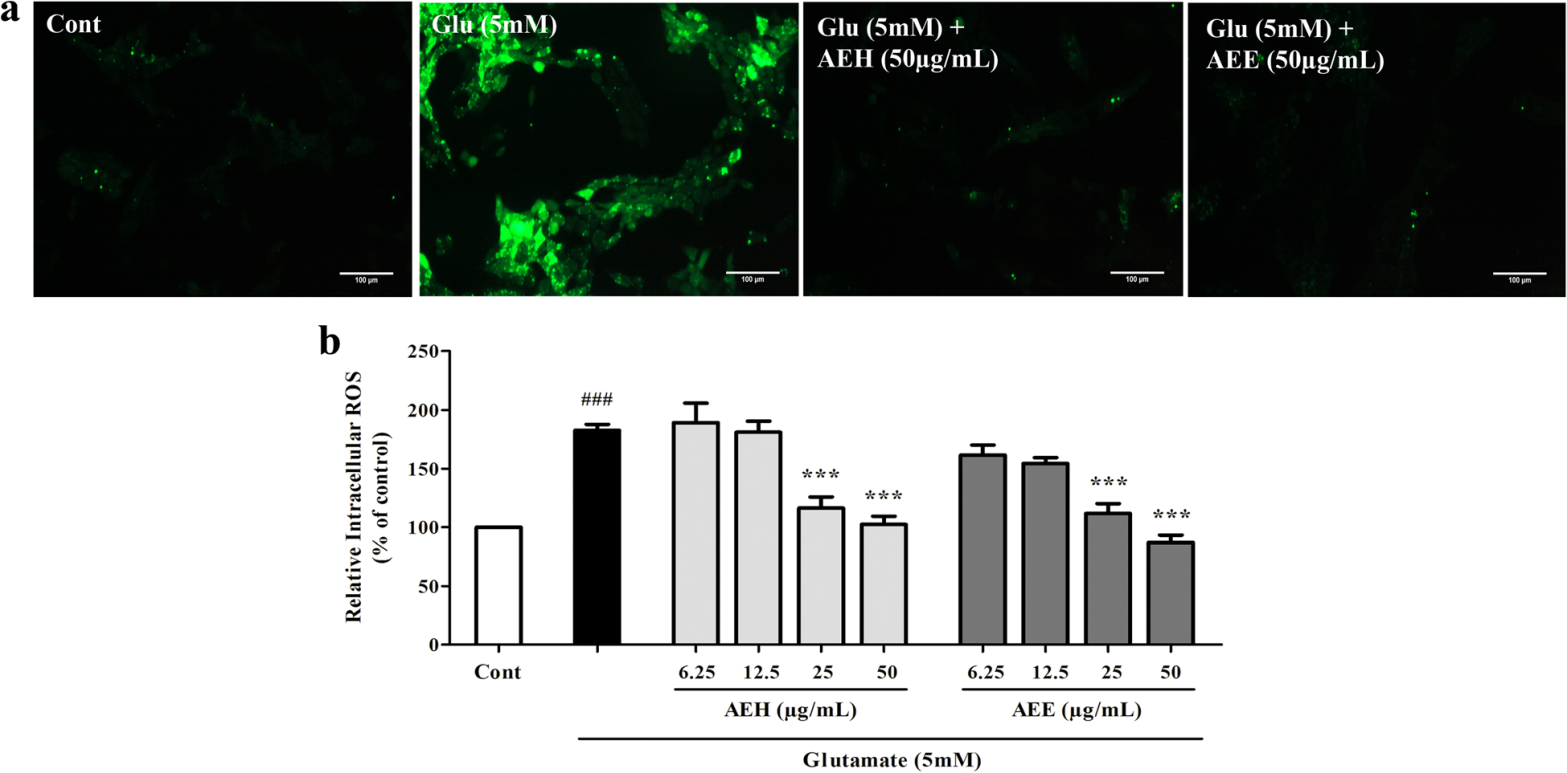
Effect of AE leaf extracts on glutamate-induced ROS accumulation in HT22 cells. (**a**) Representative confocal micrographs of DCF-derived fluorescence in untreated HT22 cells (control), or cells exposed to 5 mM glutamate alone or glutamate combined with either AEH or AEE at 50 μg/mL for 14 h. (**b**) Relative intracellular ROS levels of HT22 cells treated with 5 mM glutamate alone or with different concentrations of either AEH or AEE for 14 h, determined using a microplate reader. Data are expressed as the means ± SEM, ^*###*^*P* < 0.001 vs. control; ***P < 0.001 vs. glutamate alone

We further explored the mechanism by which AE leaf extracts protected neurons from glutamate-induced cytotoxicity. Since enhanced oxidative stress has been considered a pivotal mechanism underlying the neurotoxic action of glutamate and it is well known that increases in oxygen free radical formation trigger an AIF-mediated pathway of apoptotic cell death [14], we thus investigated whether the AE leaf extracts could attenuate ROS accumulation induced by glutamate and evaluated the antioxidant activities of the extracts in vitro. The results of the DCFH-DA assay showed that 5 mM glutamate treatment caused significantly increased intracellular ROS formation in HT22 cells, as represented by an approximately twofold higher DCF-derived fluorescence intensity relative to untreated cells (Fig. 5a and b). However, co-treatment of the cells with 50 μg/mL of either AEH or AEE was able to restore ROS production in glutamate-treated cells to a level comparable to that observed in the control cells in a dose-dependent manner (Fig. 5b). In contrast, the DPPH and ABTS assays revealed different antioxidant activities of the extracts, as shown in Table 3. The AEE exhibited a much greater capacity for radical scavenging than AEH. These data demonstrate that AE leaf extracts possess antioxidant properties and are capable of attenuating glutamate-mediated neuronal death, possibly by lowering ROS production.

**Table 3:** Free radical scavenging activities of AE leaf extracts

Sample	DPPH scavenging assay	ABTS scavenging assay		
	%Radical Scavenging activity (of 1 mg/mL extract)	mg VCEAC/g dry weight sample	%Radical Scavenging activity (of 1 mg/mL extract)	mg VCEAC/g dry weight sample
Leaf Hexane (AEH)	1.65 ± 0.17	2.44 ± 0.31	7.29 ± 1.36	4.54 ± 0.87
Leaf Ethanol (AEE)	58.46 ± 0.76	65.60 ± 1.21	89.98 ± 6.83	72.01 ± 1.02

Values are expressed as mean ± SD (*n* = 3)

### Role of Nrf2 in AE leaf extract-mediated neuroprotection against glutamate-induced oxidative toxicity

**Fig. 6:**
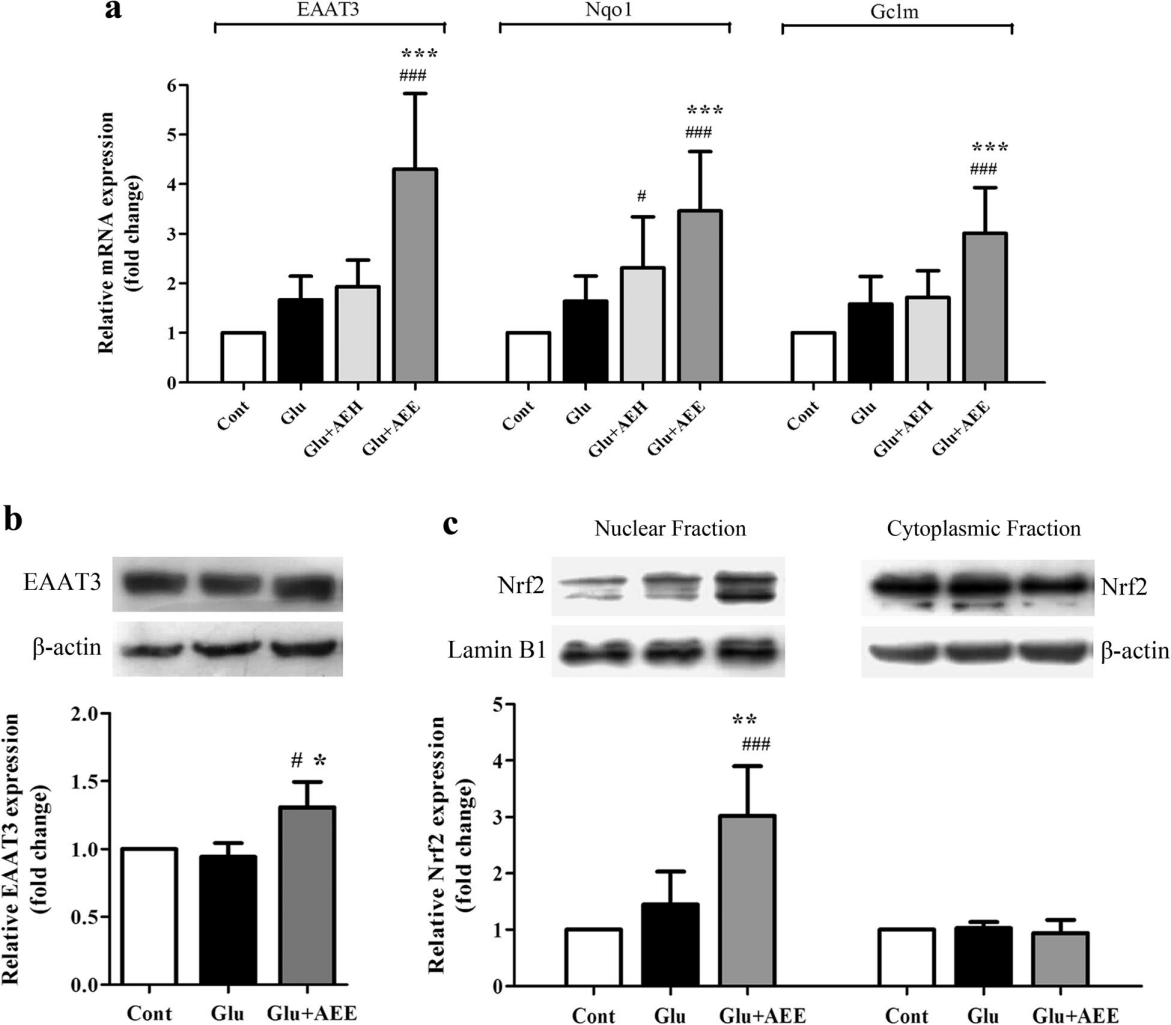
Effect of AE leaf extracts on expression of Nrf2-regulated antioxidant genes in glutamate-treated HT22 cells. (**a**) Quantitative real-time RT PCR analysis of EAAT3, Nqo1, and Gclm mRNA expression in untreated HT22 cells (control) or cells exposed to 5 mM glutamate alone or glutamate combined with either AEH or AEE at 50 μg/mL for 20 h. β-actin served as an internal control to normalize the mRNA expression levels. (**b**) Western blot analysis of EAAT3 protein in whole cell lysates isolated from untreated HT22 cells (control) or cells exposed to 5 mM glutamate alone or glutamate combined with 50 μg/mL AEE for 22 h. (**c**) Western blot analysis of Nrf2 protein in nuclear and cytoplasmic fractions isolated from HT22 cells exposed for 1 h to the similar treatment conditions as in (**b**). Lamin B1 and β-actin were used as endogenous loading controls to normalize the protein expression levels from nuclear and cytoplasmic fractions/whole cell lysates, respectively. Data are expressed as the means ± SD, ^*#*^*P* < 0.05, ^*###*^*P* < 0.001 vs. control; *P < 0.05, **P < 0.01, ***P < 0.001 vs. glutamate alone

We further elucidated the mechanism of AE leaf extract in antioxidant-mediated neuroprotection against glutamate-induced toxicity. It is well known that the induction of the Nrf2/antioxidant response element (ARE) signaling pathway is a major mechanism of cellular protection against oxidative stress by controlling the expression of antioxidant-related genes whose protein products are involved in the elimination of free radicals [36, 37]. Therefore, we examined the effect of AE leaf extracts on the Nrf2 signaling pathway by using real-time reverse transcription (RT) PCR and Western blot analysis. Our results revealed that 50 μg/mL AEE significantly increased the mRNA expression levels of antioxidant-related genes under Nrf2 regulation, including excitatory amino acid transporter 3 (EAAT3), NAD(P)H:quinone oxidoreductase (Nqo1), and glutamate-cysteine ligase modifier subunit (Gclm), by approximately 2- to 4-fold over controls and samples treated with 5 mM glutamate, whereas exposure to glutamate and AEH or to glutamate alone did not result in a significant difference in their expression (Fig. 6a). These findings were also correlated with a significant increase in protein expression of EAAT3, which was confirmed by Western blots of AEE-treated cells (Fig. 6b). Thus, we next investigated whether AEE could activate transcription factor Nrf2. We found that AEE treatment caused rapid Nrf2 accumulation in the nucleus of glutamate-exposed HT22 cells, while there was no alteration in the cytoplasmic level. After an hour-long exposure of HT22 cells to 5 mM glutamate and 50 μg/mL of AEE, the nuclear Nrf2 level was significantly elevated by 3- and 2-fold the level of the control and glutamate alone groups, respectively (Fig. 6c), indicating activation of Nrf2 by AEE. Taken together, the above results suggest that AE leaf extract protected neurons from the cytotoxic effect of glutamate, possibly by effective activation of transcription factor Nrf2, promoting the expression of downstream antioxidant-related genes of the Nrf2/ARE signaling pathway.

## Discussion

Neurodegenerative diseases are a group of disorders that occur as a result of chronic and progressive degeneration of neurons in the brain areas specific for each disorder, such as Alzheimer’s disease (AD), Parkinson’s disease (PD), Huntington’s disease (HD), multiple sclerosis (MS), and amyotrophic lateral sclerosis (ALS). Among a variety of neurodegenerative diseases, AD is the most prevalent disorder in the elderly. AD accounts for approximately 60–80% of all dementia cases and is now in the top 10 causes of death in the United States (U.S.) [38]. Moreover, the number of AD patients is rising dramatically every year, partly owing to a growing aging population worldwide [39]. The therapeutic action of most of the U.S. Food and Drug Administration (FDA)-approved AD drugs is to inhibit the enzyme acetylcholinesterase (AChE), which may not be effective in preventing or curing this kind of disease. Therefore, the development of an alternative treatment strategy for AD by focusing on other targets is promising and urgently needed for the management of neurodegenerative diseases in the near future. Currently, traditional plant-based remedies, especially in the Ayurveda system, have attracted the attention of society as well as the scientific world for their potential to treat several chronic and uncured illnesses, including neurodegenerative disorders [40–43]. The benefits of plant-derived compounds over synthetic drugs include the ease of availability at low cost and the comparative safety, especially for patients with long-term medication treatment.

Dysregulation of glutamatergic neurotransmission has been implicated as a critical contributor to various neurodegenerative diseases [11, 44–47]. Thus, several recent studies have drawn on the search of new drugs for neurodegenerative disorders by targeting this neurotransmitter. In the central nervous system (CNS), glutamate neurotoxicity is commonly mediated by two major pathways; receptor-dependent (excitotoxicity) and non-receptor-mediated (oxidative toxicity or oxytosis) pathways [35, 48]. Indeed, oxidative stress is involved not only in oxytosis but also in the mechanism of excitotoxicity, in which high intracellular Ca^2+^ influx caused by overstimulation of glutamate receptors could eventually contribute to excessive ROS production [48]. However, directly inhibiting glutamate receptors may not be a suitable approach as these receptors are important for maintaining normal brain functions [49]. Unsuccessful treatment of AD patients with mild to moderate symptoms by memantine, an antagonist of the N-methyl-D-aspartate (NMDA)-type glutamate receptor, further supports that oxidative stress, rather than receptor stimulation, plays a key role in the pathogenesis of neurodegenerative diseases [50]. Interestingly, a previous clinical trial has shown a significant beneficial efficacy of the well-known, powerful antioxidant vitamin E in comparison with memantine for mild to moderate AD cases [51]. Therefore, in this present study, we exclusively focused on the oxidative toxicity pathway in examining the cytotoxic responses to glutamate.

In researching glutamate-mediated toxicity, various cell lines have been used as model systems, each originating from different brain areas and exhibiting distinct responses to glutamate [52]. In comparison with other models, the HT22 cell line serves as an appropriate model system for mechanistic studies of oxidative stress-mediated neuronal injury induced by glutamate. Since the HT22 cells do not express the NMDA type of glutamate receptor, toxicity in this model occurs mainly through oxytosis. In this work, our experiments were performed using a high concentration of glutamate (5 mM), which caused a 50% reduction in cell viability relative to a previous report [33]. The present study also confirmed increased ROS generation and AIF nuclear translocation as the mechanism of cell death in this model. Furthermore, the majority of dead cells observed in this study, likely in the late stage of apoptosis after glutamate treatment for 18 h, is consistent with the previous finding that glutamate-induced apoptotic cell death was relatively late, ranging from 16 to 24 h post-treatment [35].

Although AE has long been used in traditional remedies, there are unexpectedly few scientific reports regarding its therapeutic usages, particularly for anti-aging and anti-neurodegeneration. This study provides the first scientific evidence on its neuroprotective activity. In this present work, we found that both AE hexane (AEH) and ethanol (AEE) leaf extracts ameliorated the cytotoxic effects of glutamate in neurons, supporting the historical use of AE as a neurotonic agent [27]. The protective mechanism of AEE against glutamate-induced neuronal cell death could be through lowering intracellular ROS levels, inhibition of nuclear AIF translocation, and activation of the Nrf2/ARE pathway. Moreover, the alteration in Nrf2 levels was observed only in the nucleus after treatment (Fig. 6c), indicating that there might be an increase in both Nrf2 expression and nuclear translocation processes. Unlike AEE, AEH showed lower free radical scavenging activity and did not increase transcription levels of Nrf2-regulated antioxidant genes. The exact mechanism of AEH to mediate neuronal protection is still unclear, and requires further investigation. A limitation of this study is that the effect of AEE upon Nrf2 activation was not directly elucidated by suppressing Nrf2 transcriptional activity using either small interfering RNA or an inhibitor. However, as Nrf2 is a central controlling factor required for mediating positive regulation of NQO1 and GCLM gene expression [53, 54], the observation that elevated mRNA levels of NQO1 and GCLM occurred upon AEE treatment support possible involvement of Nrf2 in the protective effect of AEE.

The majority of previously reported phytochemical components in AE are polyphenolic in structure [23, 28], which is generally known to possess strong antioxidant activity [55, 56]. In agreement with those previous studies, our results of total flavonoids and phenolic contents support the presence of those compounds in AE leaf extract. Additionally, the LC-MS analysis (Table 2) revealed 5 bioactive components previously reported in this plant, which includes adenosine, vicenin-2, verbascoside, isoversbacoside, and leucosceptoside A. Among these compounds, verbascoside (or acteoside) is a molecule of interest, as it has been shown to have antioxidant functions and protective activities in different cell models of neurodegeneration [30, 31, 57–61]. It is noteworthy that this compound also exerts a beneficial effect on cognition and memory enhancement [32, 62, 63]. Furthermore, we proposed 6 additional compounds, namely, betaine, verbacoside, skimmin, loliolide, andrographolide, and α-linolenic acid, which were identified according to m/z values against the database and selected due to either earlier reports of their antioxidant defense function or their potential neuroprotection roles [64–72]. Nevertheless, due to the complexity of crude extracts and limitations of the single mass analysis, all compounds proposed here need to be confirmed using other identification techniques, such as quantitative HPLC or liquid chromatography-tandem mass spectrometry (LC-MS/MS). In addition, the neuroprotective activity of the AE components should be re-evaluated later both individually and in combination.

## Conclusion

**Fig. 7:**
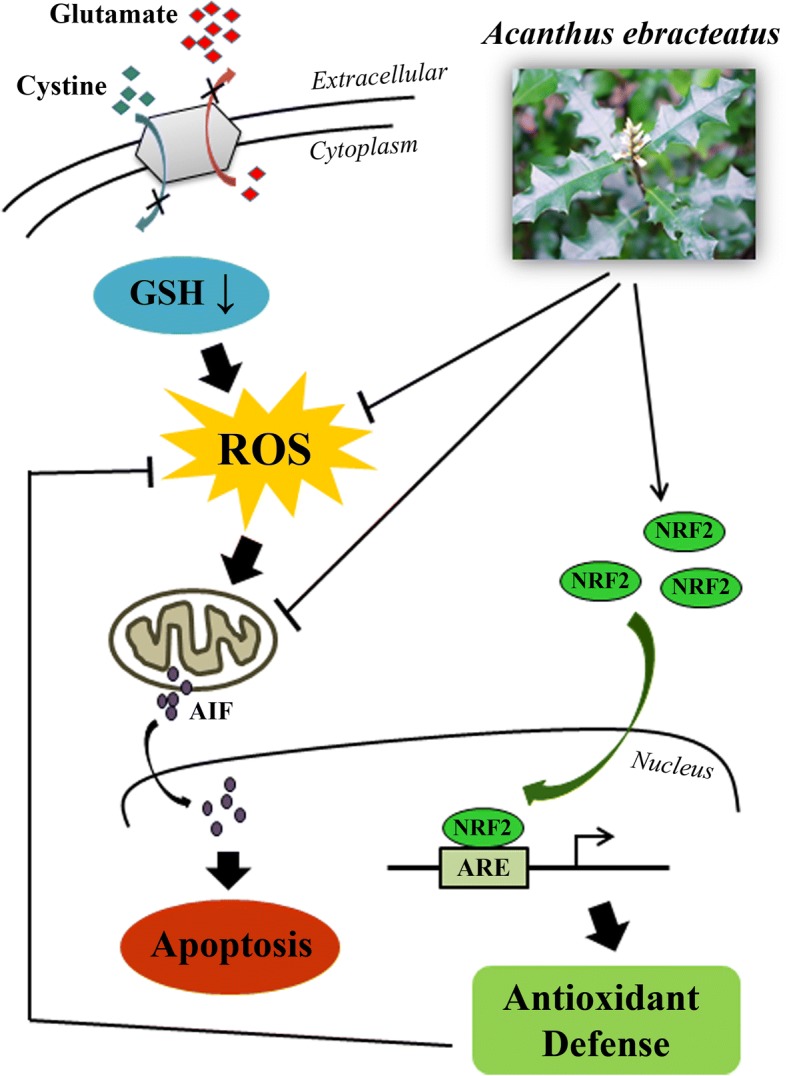
Schematic diagram showing the proposed mechanism underlying neuroprotective effect of AE leaf extract against glutamate-induced oxidative toxicity. AE leaf extract provides neuronal cell protection against oxidative stress-mediated apoptosis induced by excessive extracellular glutamate by suppressing ROS formation, inhibiting AIF translocation into the nucleus, and activating the Nrf2/ARE signaling pathway

In summary, our results demonstrated for the first time that the extract of AE leaves protects hippocampal neurons from glutamate-induced oxidative toxicity. We showed that the mechanism of neuroprotective action of AE is mediated by inhibition of the AIF-mediated apoptotic pathway and by attenuation of ROS accumulation, likely through the activation of Nrf2 antioxidant system (Fig. 7). Thus, AE may have a protective effect against neurodegenerative diseases, as well as other oxidative stress-associated disorders, due to its profound effect on the Nrf2/ARE pathway.

## Abbreviations

ABTS: 2,2′-Azino-bis(3-ethylbenzothiazoline-6-sulfonic acid) diammonium salt
AD: Alzheimer’s disease
AE: *Acanthus ebracteatus* Vahl
AEE: Ethanolic extract of *A. ebracteatus* leaves
AEH: Hexane extract of *A. ebracteatus* leaves
AIF: Apoptotic-inducing factor
DAPI: 4′,6-diamidino-2-phenylindole
DMSO: Dimethyl sulfoxide
DPPH: 2,2-Diphenyl-1-picrylhydrazyl
EAAT3: Excitatory amino acid transporter 3
GAE: Gallic acid equivalent
GCLM: Glutamate cysteine ligase complex modifier subunit
GSH: Glutathione
H_2_DCFDA: 2′, 7′-dichlorodihydrofluorescein diacetate
LC-MS: Liquid Chromatography-Mass Spectrometry
LDH: Lactate dehydrogenase
MTT: 3-(4,5-dimetylthiazol-2-yl)-2,5-diphenyltetrazoliumbromide
NMDA: N-methyl-D-aspartate
NQO1: NAD(P)H:quinone oxidoreductase 1
Nrf2: nuclear factor erythroid 2-related factor 2
PI: Propidium iodide
QE: Quercetin equivalent
ROS: Reactive oxygen species
System Xc^−^: Cystine/Glutamate antiporter
